# Mouse models of aneuploidy to understand chromosome disorders

**DOI:** 10.1007/s00335-021-09930-z

**Published:** 2021-11-01

**Authors:** Justin Tosh, Victor Tybulewicz, Elizabeth M. C. Fisher

**Affiliations:** 1 The Francis Crick Institute, London, NW1 1AT UK; 2 Department of Immunology and Inflammation, Imperial College, London, W12 0NN UK; 3 Department of Neuromuscular Disease, University College London, Queen Square, London, WC1N 3BG UK; 4 Queen Square Motor Neuron Disease Centre, Institute of Neurology, University College London, Queen Square, London, WC1N 3BG UK

## Abstract

An organism or cell carrying a number of chromosomes that is not a multiple of the haploid count is in a state of aneuploidy. This condition results in significant changes in the level of expression of genes that are gained or lost from the aneuploid chromosome(s) and most cases in humans are not compatible with life. However, a few aneuploidies can lead to live births, typically associated with deleterious phenotypes. We do not understand why phenotypes arise from aneuploid syndromes in humans. Animal models have the potential to provide great insight, but less than a handful of mouse models of aneuploidy have been made, and no ideal system exists in which to study the effects of aneuploidy per se versus those of raised gene dosage. Here, we give an overview of human aneuploid syndromes, the effects on physiology of having an altered number of chromosomes and we present the currently available mouse models of aneuploidy, focusing on models of trisomy 21 (which causes Down syndrome) because this is the most common, and therefore, the most studied autosomal aneuploidy. Finally, we discuss the potential role of carrying an extra chromosome on aneuploid phenotypes, independent of changes in gene dosage, and methods by which this could be investigated further.

**Supplementary Information:**

The online version contains supplementary material available at 10.1007/s00335-021-09930-z.

## What is aneuploidy?

### Definition of aneuploidy

Eukaryotes organise their genomes into chromosomes, with each organism having its own specific karyotype originally defined by the number and appearance of the chromosomes in the nucleus. Most eukaryotes have two of each chromosome (diploid) and therefore carry two sets of genetic information. Aneuploidy is the state in which a cell does not contain an exact multiple of the haploid chromosome count (one set of the individual chromosomes), leading to an unbalanced genomic state. Polyploid cells carry more than two full sets of the haploid chromosome count (triploid, tetraploid, etc.). Polyploidy in all cells of an organism is seen in some vertebrates, such as salmon and *Xenopus leavis*, though not most. However, tissue-specific polyploidy is common, for example, in hepatocytes, megakaryocytes and the placenta in humans. Polyploidy is often found in flowering plants, leading to a balanced increase in genetic information which can increase evolutionary fitness and lead to speciation (Rieseberg and Willis 2007).

Aneuploidies in humans are divided into two major categories, depending on whether the extra or missing chromosome(s) from the haploid karyotype is one of the 22 *autosomal chromosomes* or X or Y *sex chromosomes*. Partial aneuploidies of both the autosomes and sex chromosomes also exist wherein only part of a chromosome is missing or duplicated. This can lead to an unbalanced genetic state and, because partial aneuploidies tend to be single cases, experiments to investigate them are rarely undertaken. However, panels of clinically and genetically assessed partial aneuploidy individuals with deficits in the same chromosome, have been set up to try to identify regions of dosage sensitive genes (Shapiro 1999), but such attempts are confounded by limited clinical records and phenotypic variability of patients. All forms of aneuploidy cause deleterious effects such as developmental defects, spontaneous abortions and increased susceptibility to cancer (Chunduri and Storchová 2019).

### Incidence and causes of aneuploidy

In humans, approximately one-third of all miscarriages are caused by aneuploidy and it is estimated that 10–30% of all fertilised eggs are aneuploid prior to implantation (Hassold and Hunt 2001). It has been known for some time that advanced maternal age at conception greatly increases the risk of aneuploidy in conceptuses. Among women under the age of 25, only 2% of pregnancies have been detected as trisomic, but in women over 40 years of age this rises to approximately 35% (Antonarakis et al. 1992; Hassold and Hunt 2001; Nagaoka et al. 2012).

Increased risk of aneuploidy with advanced maternal age probably arises from a variety of different causes (Mikwar et al. 2020). When cells divide through mitosis or meiosis, the chromosomes are segregated between the daughter cells or gametes in a highly organised manner that relies on precise organisation of the microtubule network and spindle assembly checkpoint pathway to proceed correctly (Hassold and Hunt 2001). Down syndrome arises from having three copies, trisomy, of human chromosome 21 (Hsa21). In cases of trisomy 21 pregnancies from mothers of advanced age, chromosome segregation errors have been detected derived from both meiosis I and meiosis II, although the cause of these errors has not been fully elucidated. Current evidence suggests many factors are involved including failure of recombination in prophase of meiosis I, deterioration of chromosome cohesion linked to oocyte age and mitochondrial dysfunction. Aneuploidy can also arise from errors in mitosis and so may cause somatic mosaicism, depending on early the mitotic error occurs—for example, up to 5% of Down syndrome cases may be mosaics for euploid and aneuploid (i.e. trisomy Hsa21) cells (Thorpe et al. 2020). For a comprehensive review of likely causes of increased risk of aneuploidy with advanced maternal age, please read (Mikwar et al. 2020).

Ultimately, errors in these processes can cause non-disjunction wherein the chromosomes fail to separate, leaving one of the daughter cells with an unbalanced set of chromosomes (Compton 2011). In embryos, aneuploidy can lead to spontaneous abortion; however, studies in mice show that if only a proportion of embryonic cells are aneuploid (mosaic), then the embryo may self-correct towards euploidy from the blastocyst stage onwards. Aneuploid cells are depleted via a combination of apoptosis and faster growth of diploid cells. Such embryos were found to be viable with full developmental potential, but we do not know whether these corrective processes exist in human embryos with mosaic aneuploidy and how frequently this phenomenon occurs (Bolton et al. 2016). Aneuploidy syndromes can also be caused by Robertsonian translocations (RTs) of the long arms of the acrocentric human chromosomes (Hsa13, 14, 15, 21 and 22) to form dicentric fusion chromosomes. For example, the most common RTs involve fusion of Hsa13 with Hsa14 and Hsa14 with Hsa21. In fact, between 3 and 4% of people with DS have inherited a chromosome with an RT that includes the long arm of Hsa21 (Shin et al. 2010). Because overall genetic content is still balanced, the initial heterozygous carrier may not exhibit any deleterious phenotypes, however, any progeny who inherit the fusion chromosome will carry an unbalanced set of genes and could exhibit aneuploid-like phenotypes without carrying an extra chromosome (Poot and Hochstenbach 2021).

### Consequences of aneuploidy on cell physiology

Aneuploidy has diverse consequences for cell physiology. Notably, it leads to an increase in the expression or “dose” of genes in trisomies or a decrease of gene dose in monosomies (loss of a chromosome) for loci on the aneuploid chromosome (Dürrbaum and Storchová 2016). The result of over and under-expressed genes in aneuploidy is called the “gene-dosage” effect, which refers to the direct consequences of overexpressed genes *and* to the downstream consequences of this overexpression on other pathways in the cell (Antonarakis et al. 2020; Pritchard and Kola 1999). We will consider the consequences of gene dosage in section “Aneuploidy and gene dosage in clinical phenotypes: what is the current evidence?”.

Aneuploidy can alter the proliferative characteristics of cultured cells. For example, trisomy of chromosome 8 in mouse embryonic stem cells is commonly observed during culture, conferring an advantage for proliferation and a reduction in the ability to generate germline competent mice (Liu et al. 1997). In human stem cells, trisomy 12 has a similar effect, significantly increasing proliferation rate and also conferring a transcriptomic profile similar to germ cell tumours (Ben-David et al. 2014). Conversely, aneuploidies can cause a proliferative disadvantage depending on cell type and chromosome identity. In both human and yeast aneuploid lines, various trisomies cause a reduction in growth and delay in the G1 and S phases of the cell cycle. This could be due to a reduction in fitness caused by gene-dosage effects of the aneuploid chromosome, although some evidence points towards a delay in the accumulation of cyclins necessary to progress in the cell cycle (Segal and McCoy 1974; Stingele et al. 2012; Torres et al. 2007). Additionally, cell lines engineered to be trisomic for chromosome 8 or chromosome 22 both show decreased proliferation and genomic instability (Ariyoshi et al. 2016). Genomic instability may be a hallmark of aneuploidy. Passerini et al. found that the gain of a single chromosome in cultured human cells increases the occurrence of DNA damage and causes replication stress wherein progression through S-phase of cell replication is slowed. These effects were independent of chromosome identity and were associated with accumulation of chromosome rearrangements across the entire genome (Passerini et al. 2016). It is important to note that these effects have yet to be observed in aneuploid whole organisms so further study is required.

Aneuploidy can also have a profound effect on nuclear topology in affected cells. The presence of an extra chromosome disrupts nuclear morphology into irregular and contorted shapes compared to wildtype cells with a concomitant increase in nuclear volume. There is a linear relationship between the number of genes encoded on the aneuploid chromosome and increases in the volume of nuclei (Hwang et al. 2019). Hwang et al. detected an increased dependence on long-chain bases which are synthesised into lipids critical for maintaining nuclear morphology in aneuploid human fibroblasts, seemingly independent of aneuploid chromosome identity. By increasing levels of long-chain bases, they rescued the nuclear membrane phenotype, suggesting that targeting lipid biosynthesis pathways may represent a common therapeutic strategy for multiple aneuploid syndromes (Hwang et al. 2019).

Cells with aneuploidies have altered metabolic properties compared to euploid controls. When Williams et al. measured the production of the glutamine metabolite ammonium in mouse fibroblast lines with various trisomies, all showed a significant increase after extended culture. Additionally aneuploid yeast lines showed an increase in turnover due to increased synthesis and degradation of superfluous proteins encoded on the extra chromosome (Torres et al. 2007; Williams et al. 2008).

## What are the human aneuploidies?

### Autosomal aneuploidies

In humans, the most common autosomal aneuploidy (AA) is trisomy of human chromosome 16 (Hsa16), which in its complete form is incompatible with life and leads to approximately 6% of all miscarriages between 8 and 15 weeks of gestation (Benn 1998). Mosaic cases of trisomy 16 in which only a proportion of cells in the body contain an extra copy of Hsa16 are generally tolerated with developmental issues depending on the level of mosaicism (Sparks et al. 2017).

All human autosomal trisomies are incompatible with life, and cause spontaneous abortion, other than three: trisomy 13, 18 and 21. Trisomy 21 causes Down syndrome (DS), the most common AA with, for example, an incidence of 12.6 in 10,000 live births in the United States (de Graaf et al. 2016). DS is characterised by a wide-range of clinical features, which may be considered *invariable*, occurring in everyone, with different severity, or *variable*, occurring with incomplete penetrance in a subset of people with DS. Invariable features include learning and memory deficits, characteristic facial dysmorphology, and a high-risk of early onset Alzheimer’s-type dementia (McCarron et al. 2017; Wiseman et al. 2015). Variable features include congenital heart defects, increased incidence of blood cancers, and an elevated risk of autoimmune disease (Antonarakis et al. 2020).

Trisomy 18 causes Edwards syndrome (ES) and is the second most common AA in humans occurring in around 1 in 6000 live births, but with an overall incidence estimated to be closer to 1 in 2500 including fetal loss and pregnancy termination after prenatal diagnosis (Cereda and Carey 2012; Root and Carey 1994). Edwards syndrome is characterised by severe prenatal growth deficiency, characteristic facial features and a distinctive hand posture with overlapping fingers. Additionally, babies with Edwards syndrome commonly present with heart malformations (Cereda and Carey 2012).

Trisomy 13 is the cause of Patau syndrome (PS), which is the third most common AA affecting around 1 in 10,000 live births and causing phenotypes such as slow prenatal development, holoprosencephaly (failure of the forebrain to divide correctly), heart defects and cleft palate. Patau and Edwards syndrome both have a mortality rate of over 90–95% before one year after birth (Rasmussen et al. 2003).

Autosomal monosomy is another form of human aneuploidy; loss of any of the autosomes is not compatible with life (Bunnell et al. 2017).

### Sex chromosome aneuploidies

Compared with autosomal aneuploidies, sex chromosome aneuploidies (SCA) are better tolerated in humans. The five most commonly detected are Klinefelter syndrome (XXY, 1:750 live births), Turner syndrome (XO, 1:2500 live births), trisomy X (XXX, up to 1:1000 live births), XYY (up to 1:1000 live births, and XXYY (1:20,000 live births). All SCAs are underdiagnosed because of their comparatively mild phenotypes (Skuse et al. 2018). This is most likely due to the random inactivation of all but one X chromosome even in cells which carry more than two X chromosomes, such as in trisomy X or XXXX (Migeon et al. 2008). Despite this, X-inactivation is not complete, approximately 15% of human X-linked genes are biallelically expressed from the pseudoautosomal regions on the X chromosome and specific genes outside these regions can be transcribed at limited levels in a tissue-dependent manner (Berletch et al. 2011). Loss of the X chromosome leading to a karyotype of 45, Y (i.e. no X chromosome) is not compatible with life, which is perhaps not surprising as the human X chromosome makes up ~ 5% of the genome. A karyotype of 45, X, i.e. with no second sex chromosome, results in Turner syndrome, which manifests with short stature, infertility and a range of other variable characteristics, but is compatible with life (Gravholt et al. 2019).

Here, we do not discuss SCAs in depth, but confine our review to the autosomal aneuploidies as again, this is one of the most developed areas of mouse modelling of these chromosomal disorders.

### Aneuploidies in cancer

While not the focus of this review, aneuploidies are often observed in cancer cells. Cancer cells frequently demonstrate chromosomal instability (CIN) leading to ongoing karyotypic changes which can confer selective advantages and contribute to cancer evolution. Whole chromosome and segmental aneuploidies are proposed to facilitate fast, wholesale alterations in cancer phenotype, and contribute to tumour heterogeneity. Please see (Sansregret and Swanton 2017) for a comprehensive review of the role of aneuploidy in cancer.

## Aneuploidy and gene dosage in clinical phenotypes: what is the current evidence?

Whole-chromosome aneuploidy has diverse and profound consequences for multiple aspects of cell physiology such as proliferation, genomic stability and tumorigenesis. From studies of the effects of copy number variation, and of aneuploidy syndromes, it is clear that for some genes or alleles, dosage critically affects phenotype although in most cases we do not know which genes are the culprits. However, a question that remains entirely unaddressed in mammalian systems in vivo, and the focus of this review, is the possible role(s) of aneuploidy itself—i.e. the state of having an extra chromosome—in producing clinical phenotypes. No studies yet address this question directly in mammalian model systems but we can make some inferences from the current literature and make suggestions for new model systems which may facilitate work in this area.

### An historical view of aneuploidy effects

**Fig. 1:**
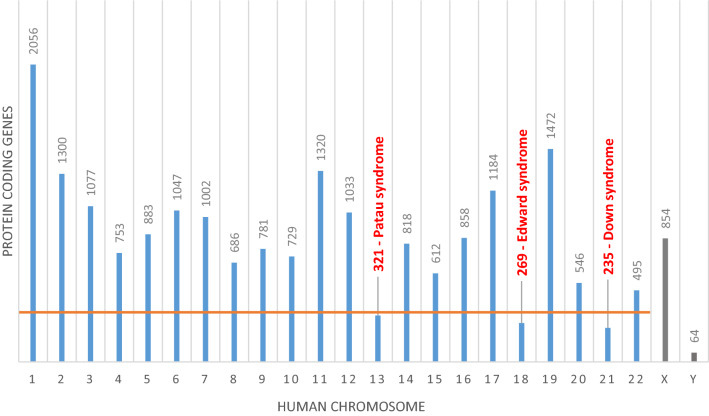
Graph showing the number of known protein coding genes on each human chromosome. Trisomies of chromosomes with bars below the orange line are compatible with life. All gene totals taken from Ensembl genome build GRCh38.p13. Sex chromosomes are shown in grey

An older viewpoint in favour of aneuploidy per se being causal for the clinical features of chromosome disorders is related the number of shared phenotypes seen in individuals who have the autosomal trisomies that are compatible with life (i.e. trisomy Hsa13, 18, 21), despite a completely different gene content on the trisomic chromosomes. For example, babies with DS, PS and ES all present with developmental abnormalities of the nervous system, overall developmental delay, and cardiovascular and craniofacial defects (Pai et al. 2003). However, despite these similarities it is also clear (1) that each syndrome is entirely distinct from the other, in terms of the specific developmental anomalies that arise, including lifespan, and (2) that the number of expressed genes encoded on each chromosome has a direct relationship with the severity of the syndrome, with trisomy 13 being more severe than trisomy 18, and both of these more severe than trisomy 21 (Fig. 1).

Trisomy 13, 18 and 21 can be classified together as the only AAs compatible with live birth in humans although accompanied by various pathologies, with all other AAs causing embryonic lethality. These trisomies are sometimes referred to as ‘subviable’ as in each case only a percentage of individuals come to term, because autosomal trisomy is so deleterious; for example, estimates suggest up to 43% of trisomy 21 fetuses may miscarry (Morris et al. 1999) In fact chromosomal abnormalities including aneuploidies are found in chorionic villi samples from approximately 50% of first trimester spontaneous abortions (Eiben et al. 1990). The causative mechanism of miscarriage of aneuploid foetuses is unclear, however it is likely to involve a combination of factors related to the specific alleles found on the aneuploid chromosome, the number of genes the chromosome encodes for and cellular processes which detect errors in DNA replication and cell division (Chunduri and Storchová 2019; Torres et al. 2008).

Therefore, although some broad phenotypes are shared between the trisomies compatible with life, there are sufficient differences to indicate that most of the clinical features arise from having three doses of specific genes on each chromosome. However, this still leaves the role of aneuploidy per se, unknown.

### Could aneuploidy play a role, in addition to gene dosage effects, in clinical features of trisomy?

In aneuploid cells, genes on the extra chromosome are generally transcribed and translated, leading to roughly a linear relationship between gene copy number, and mRNA and protein expression, which for some genes causes gene-dosage derived phenotypes. These genes are defined as ‘dosage-sensitive’ and are proposed to be the primary cause of aneuploid syndromes (Antonarakis et al. 2020; Ishikawa et al. 2017; Khan et al. 2013; Stingele et al. 2012).

It is also possible that aneuploidy per se could also play a role in inducing phenotypes. There are only a handful of properly-controlled studies which directly test this hypothesis, but a notable piece of evidence regarding a role for aneuploidy was provided by Torres et al. in 2007, working with yeast models (Torres et al. 2007): to exclude gene dosage effects of aneuploidy, the authors generated several yeast artificial chromosomes (YACs) carrying different amounts of human DNA sequence. These were introduced into haploid yeast cell lines, creating clones carrying an extra chromosome without any transcribed yeast genes, therefore controlling for gene-dosage specific phenotypes. Nevertheless, when accounting for doubling time using phosphate-limited culture conditions, the presence of a YAC consistently affected gene expression from *other* chromosomes and the yeast line with the largest human DNA containing YAC (~ 1.6 Mb) showed slightly delayed entry into the cell cycle (Torres et al. 2007). This interesting study was conducted in yeast cells but it provides clues to possible mechanisms that could also apply to the human aneuploidies. The smallest human chromosome is Hsa21 which is approximately 46 Mb in length, much *larger* than the YACs used in this study, so it would be useful to perform similar experiments in mammalian cells to see if the underlying unknown mechanisms still apply.

Presumably, the introduction of noncoding DNA is unlikely to affect the fitness of cells as much as additional DNA that can be transcribed and translated. However, while many investigations address the role of gene-dosage in aneuploid phenotypes in mammals, most studies do not control for, or investigate, aneuploidy per se. A rare example in which the researchers shed some light on aneuploidy effects in humans came from assessing effects of different chromosome aneuploidies within the same experimental system. Passerini et al. observed that engineered aneuploid cell lines with five different trisomies consistently showed increased levels of DNA damage markers such as 53BP1 foci in pre-mitotic G1 cells across all chromosomes, i.e. the addition of single, different, extra chromosomes to human cells in culture promoted genomic instability by increasing DNA damage and sensitivity to replication stress (Passerini et al. 2016). Further investigation showed that the replicative helicase MCM2-7 was downregulated in all aneuploid lines tested and that its overexpression could partially rescue the phenotype. Why replication factors such as MCM2-7 are downregulated in response to aneuploidy remains unknown, but may be related to global transcriptional changes in RNA and DNA metabolism pathways which are observed in aneuploid cells regardless of the specific abnormal karyotype. Perturbations and deficiencies in HSP90-mediated protein folding were also found to be a general characteristic of aneuploid mammalian cells, which could impact production of MCM subunits (Donnelly et al. 2014; Stingele et al. 2012).

Finally, in considering human aneuploidy effects, we note that approximately 3% of people with DS are euploid, because they have a Robertsonian translocation of the long arm of human chromosome 21, Hsa21q, to the long arm of usually another acrocentric chromosome, most often Hsa14q. These cases are not informative for considering the role of aneuploidy in DS because, (1) gene content is not equivalent between aneuploid and euploid DS because the missing short arms from Robertsonian translocation cases carry ribosomal RNA sequences that could produce phenotypes, for example, affecting protein translation. Also, although likely few and far between, the short arms carry unique protein coding regions that presumably have some conserved function. (2) DS is highly variable and effects of aneuploidy may be subtle although notable over life, thus very large numbers of individuals with euploid and aneuploid forms of DS would need to be compared to each other to see if consistent phenotypes segregated with one form or the other—an impossible undertaking when the phenotype is hugely variable, as is the case for DS—although feasible for the tightly controlled genetics of mouse models.

In summary, data from yeast aneuploidy models showed perturbed cellular metabolism and proteostasis leading to dysfunctional quality control in protein folding (Torres et al. 2007). However, most mammalian studies address gene-dosage effects but do not have suitable controls or experimental breadth to address effects of aneuploidy itself. [For a more in-depth review of the consequences of aneuploidy we recommend a review by Chunduri and Storchova (Chunduri and Storchová 2019)]. Thus we require mammalian models to build on the work of Torres, Amon and co-authors, and investigate effects of aneuploidy separately from effects of gene dosage.

## Models of aneuploidy

Most of the currently available mouse models of aneuploidy syndromes primarily model gene-dosage abnormality, not aneuploidy itself because they do not have an extra chromosome, but instead carry duplications of sets of mouse genes orthologous to the human chromosome of interest.

In this review, we focus on Down syndrome (DS) to illustrate the creation and use of mouse models of aneuploidy, because it is the most common and widely studied autosomal aneuploidy and the most advanced disorder in terms of available model strains.

A large number of these ‘chromosome-engineered’ DS models have been generated using Cre/*LoxP* recombination to duplicate discrete syntenic regions of mouse chromosomes orthologous to Hsa21, internally within individual chromosomes. While these allow us to investigate the dosage-sensitivity of many different Hsa21 orthologues, such duplication models are not aneuploid, because they have a normal diploid chromosome number. Nevertheless, they are extremely useful tools to understand the role of gene-dosage in aneuploidies such as DS, since it is clear that over-dosage of genes and concomitant effects on gene interaction networks are key drivers of aneuploidy phenotypes (Herault et al. 2017; Torres et al. 2008).

Again, what is not clear is whether there are specific phenotypes that are caused by having an extra chromosome. Currently there are no mammalian cell or animal models which can address this question with appropriate controls. Thus it is essential that we recreate the ability to distinguish the potentially different effects of aneuploidy and gene-dosage within a mammalian system.

### Current mammalian models of aneuploidy

There are very few animal models that are aneuploid and carry an extra chromosome with genes orthologous to a human chromosome of interest. For example, there are no mouse models of ES and PS (Sheppard et al. 2012). Modelling these trisomies is complicated because Hsa18 (ES) gene orthologues in the mouse are syntenic to five distinct regions across three separate chromosomes. Similarly Hsa13 (PS) orthologues in the mouse are spread across six mouse chromosome segments. Hsa21 orthologues in the mouse are located in three syntenic regions on Mmu10, Mmu16 and Mmu17 with the largest being on Mmu16 and spanning approximately 23 Mb. To generate a comprehensive model with duplications of all relevant genes is challenging with existing techniques, especially as the numbers of regions of homology in the mouse genome rise, as for PS and ES.

The first postnatally viable mouse model of DS was the Ts65Dn strain published in 1990, which carries around 90 protein-coding Hsa21 orthologues in three copies on a supernumerary hybrid chromosome comprised of the centromere of Mmu17 (~ 10 Mb of Mmu17) and 13.4 Mb of distal Mmu16 orthologous to Hsa21 (Davisson et al. 1990; Duchon et al. 2011; Reeves et al. 1995). This strain has been thoroughly characterised and has a number of DS-like phenotypes such as craniofacial dysmorphology, heart defects and learning impairments (Costa et al. 2010; Herault et al. 2017; Reeves et al. 1995). However, because the Ts65Dn mouse also carries the three copies of the ~ 10 Mb Mmu17-derived region, which includes 35 protein coding genes that are not orthologous to genes on Hsa21, some of the gene-dosage effects seen in this strain may not relate to DS (Duchon et al. 2011; Reinholdt et al. 2011). This is particularly relevant to neurological and cognitive phenotypes as this non-Hsa21 orthologous region includes genes involved in synaptogenesis. The Ts65Dn strain has now been superseded by genetically more accurate mouse models of DS.

The Ts65Dn strain was generated by painstakingly screening for Robertsonian translocations of Hsa21 orthologous regions. It was (and still is) difficult to engineer true models of aneuploidy in mammals due to the limited techniques available. To avoid the problem of mouse Hsa21 orthologues being on three different chromosomes, two models were designed with the approach of adding Hsa21 into mouse cells, to create transchromosomic strains. A technique for transferring single chromosomes between cells has existed since the 1970s called microcell mediated chromosome transfer (MMCT) (O’Doherty and Fisher 2003). Briefly, MMCT is performed by arresting donor cells with the chromosome of interest in metaphase using a drug to inhibit microtubule spindle assembly. After prolonged treatment, surviving cells will undergo mitotic slippage and form micronuclei containing few or single chromosomes. The micronuclei can then be extruded from the cells by centrifugation in the presence of an actin polymerisation inhibitor, collected, and fused with recipient cells. If the chromosome of interest has a selectable marker then the resultant microcell hybrids can be clonally selected and expanded (Fournier and Ruddle 1977; Lugo et al. 1987). MMCT was used to try to transfer human chromosomes from human cells into mouse embryonic stem cells, which could then be injected into blastocysts using conventional transgenic techniques, to create chimeric embryos and ultimately achieve germline transmission of the human chromosome, in transchromosomic mice.

Early attempts to generate transchromosomic models carrying a human chromosome were only partially successful. Aneuploid chimeric mice carrying Hsa21 were generated by two groups between 1997 and 1999, however, the transchromosome did not transmit through the germline (Hernandez et al. 1999; Tomizuka et al. 1997). Germline transmission of a fragment of Hsa21 was successfully achieved in 2001 (Kazuki et al. 2001), but it would take until 2005 for the first transchromosomic mouse model of DS carrying a freely-segregating Hsa21 to be published: the Tc1 mouse (O’Doherty et al. 2005). Tc1 mice express human Hsa21 genes, as shown by transcriptional and protein studies, and recapitulate various phenotypes of DS including heart defects, memory and neuronal deficits and craniofacial dysmorphology (for example, Ahmed et al. 2013; Dunlevy et al. 2010; Haas et al. 2013; Hall et al. 2016; O’Doherty et al. 2005; Reynolds et al. 2010; Watson-Scales et al. 2018; Wiseman et al. 2018; Witton et al. 2015). During generation of the model however, the Hsa21 chromosome underwent a number of deletions and rearrangements such that approximately 75% of protein coding genes are present (Gribble et al. 2013). The mouse is also mosaic, on average approximately 66% of brain cells carry the Hsa21 and this percentage varies in different tissues in different animals. This is likely due to the Hsa21 transchromosome carrying a human centromere, as determined by Southern blot hybridisation with human-specific centromeric repeat probes (O’Doherty et al. 2005), which has been shown to cause chromosomal instability and loss in mouse cells (Shinohara et al. 2000).

The newest aneuploid model of DS is the TcMAC21 mouse which was engineered to carry a mouse artificial chromosome (MAC) with a nearly complete copy of Hsa21q (Kazuki et al. 2020). Importantly TcMAC21 is not mosaic for the transchromosome, likely due to the fact that the Hsa21-MAC has a mouse-derived centromere. This model is currently the most complete aneuploid model of DS, however, it still has some limitations. First, during generation of the model, a number of large-scale deletions occurred such that approximately 28% of Hsa21q is missing. Fortunately, protein-coding gene dense regions were spared and only 14 Hsa21 genes are missing from the Hsa21-MAC. While the authors did not suggest a mechanism for these deletions, it is possible that in the process of transferring the chromosome using MMCT it underwent chromothripsis (Kneissig et al. 2019). When a chromosome undergoes chromothripsis it is shattered and aberrant DNA repair processes lead to large scale rearrangements, deletions and duplications (Forment et al. 2012). Supplementary Table 1 shows the triplicated gene content of the currently available aneuploid mouse models based on Ensembl genome build GRCm39.

**Fig. 2:**
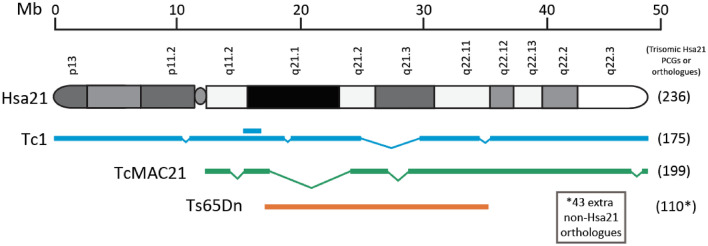
Aneuploid mouse models of DS. A schematic megabasepair ruler is shown at the top. Human chromosome 21 (p and q arms with G-banding) is shown below this. The Tc1 model is shown in blue with deletions and a duplication (double line segment) relative to Hsa21. The transchromosomic TcMAC21 is depicted in green with deletions relative to Hsa21. The Hsa21 trisomic region in TcMAC21 is incorporated into a mouse artificial chromosome with a mouse centromere. The trisomic Hsa21-orthologous region in Ts65Dn mice is shown in orange relative to Hsa21. Numbers of trisomic Hsa21 genes (or mouse orthologues) in each model are shown to the right in parentheses. The Ts65Dn model carries an extra 43 protein coding genes not orthologous to Hsa21

The Ts65Dn, Tc1 and TcMAC21 strains (Fig. 2) each give new insights into the biology of DS, however, they all have the key limitation that none control for the effect of aneuploidy, rather than gene-dosage. Recently Duchon et al. carried out a comparative study of behavioural phenotypes in several segmental duplication models of Down syndrome carrying different sets of Hsa21 orthologues in three copies (Duchon et al. 2021). When compared with the aneuploid Ts65Dn strain, the non-aneuploid mice did not show deficits in the Morris water maze and open-field test, despite very similar trisomic gene content. This could be due to the extra Mmu17 genes in the Ts65Dn not orthologous to Hsa21, or the suppressive effect of the relatively few Hsa21 orthologues that are not triplicated in Ts65Dn mice, or importantly, an effect of aneuploidy that is separate from gene dosage. Duchon et al. also determined that while overexpression of *Dyrk1a* is minimally required to cause a number of behavioural phenotypes, other genes orthologous to Hsa21 likely modify the effects. Comparisons between animals overexpressing *Dyrk1a* with different supplementary Hsa21 orthologues in three copies identified a region between *Cbr1* and *Fam3b* which rescues increased activity during open-field testing (Duchon et al. 2021).

Previous studies of the multi-genetic factors at play in DS phenotypes using segmental models give further evidence for the complex nature of aneuploidy syndromes. Lana-Elola et al. used a mapping panel of seven segmental DS mouse models to identify a “minimal critical region” of trisomic genes sufficient to cause atrio-ventricular septal defects and showed that there must be at least 2 causative genes (Lana-Elola et al. 2016).

Ideally, developing panels of aneuploid models would be an essential complement to the wide array of duplications that are currently being studied.

### Ideal models

**Fig. 3:**
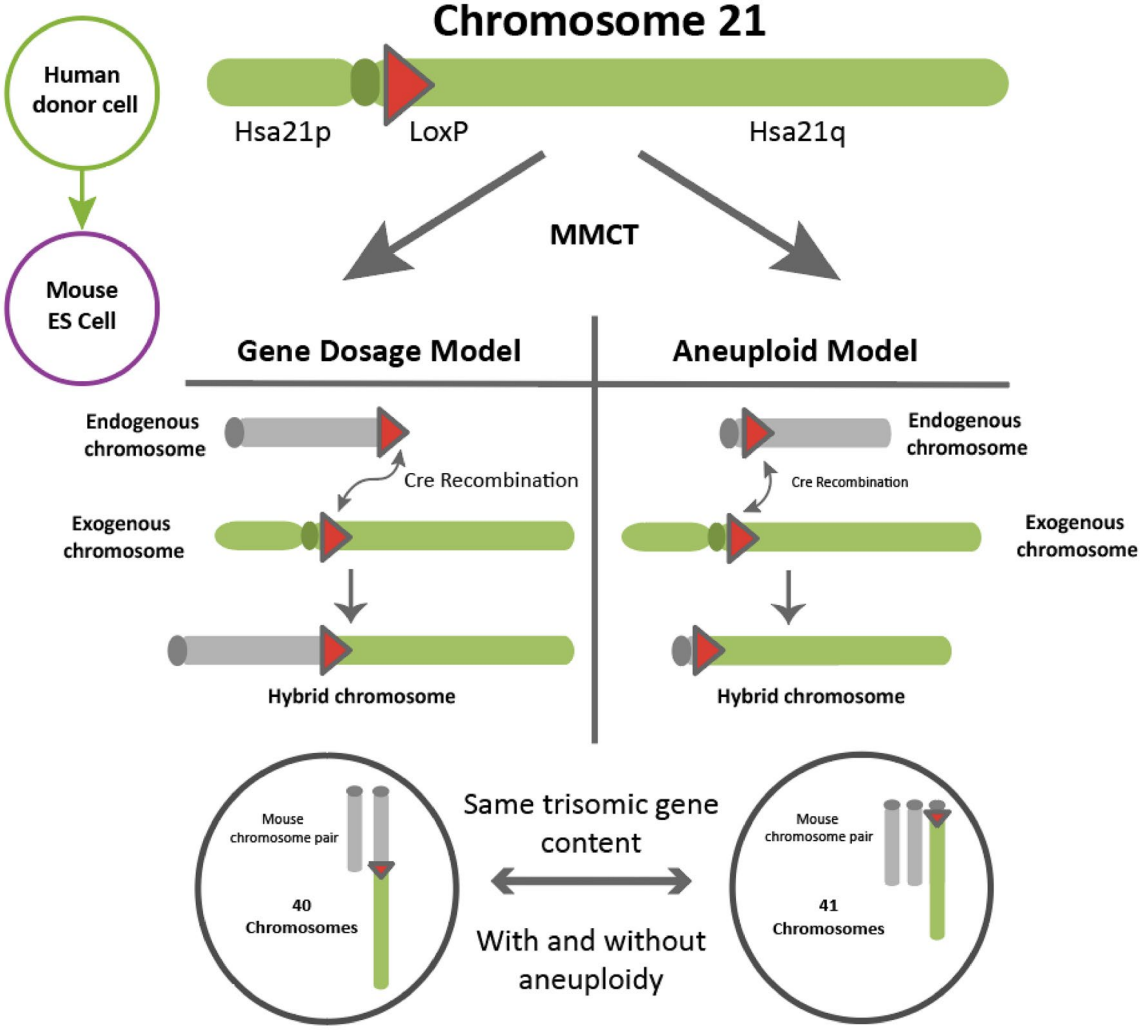
A proposed animal model system for DS (trisomy 21) consisting of two complementary models. For the gene dosage model, gene targeting is used to insert recombination sites (LoxP) into sequences close to the centromere of Hsa21 and telomere of a mouse chromosome. MMCT is used to move Hsa21 into targeted mouse embryonic stem cells. In vitro Cre expression recombines Hsa21q onto the end of the mouse chromosome. Resulting mice will have 3 copies of Hsa21 orthologues but will have a euploid chromosome count (reciprocal hybrid chromosome not shown and would not be retained). For the aneuploid model, gene targeting is used to insert recombination sites (LoxP) into sequences close to the centromere of Hsa21 and centromere of a mouse chromosome. MMCT is used to move Hsa21 into targeted mouse embryonic stem cells. In vitro Cre expression replaces the endogenous mouse chromosome arm with Hsa21q. After generation of mice and backcrossing, resulting mice will have 3 copies Hsa21 orthologues with an aneuploid chromosome count of 41 (the euploid mouse chromosome karyotype has 40 chromosomes)

The models of aneuploidy we have access to currently cannot be used to address the question of whether aneuploidy per se has a role in phenotypes seen in conditions such as DS. For this, the key criterion would be the ability to compare the phenotypic effects of gene dosage increases of aneuploidy *with* and *without* an extra chromosome. In Fig. 3 we suggest an example model system that could be created using current techniques, which would address the issue of aneuploidy. In this example, Hsa21 was chosen because Hsa21 is acrocentric and almost all protein coding genes are located on the long arm (Hattori et al. 2000), reducing the complexity of chromosome engineering required, although we note that ultimately to understand DS we need to know if Hsa21p plays a role in phenotype. Models of monosomy could also prove useful, not only for studying AA or SCA disorders with partial or full chromosome loss, but also as a tool to selectively reduce the copy number of regions/ genes which may contribute to aneuploid phenotypes.

## Future prospects

Chromosome engineering techniques give us the ability to routinely generate animal models with specific segmental duplications and/or deletions, and there are now a large number of models available to investigate the gene-dosage hypothesis, especially for DS (Herault et al. 2017). Truly aneuploid models present a greater challenge to develop and so there are fewer available for study. However, to address this, it may in future be possible to synthesise large enough DNA fragments to construct mammalian chromosomes from scratch. Researchers in the field of synthetic biology have recently replaced a number of *Saccharomyces cerevisiae* chromosomes with custom-designed synthetic chromosomes. However, this was completed in multiple small steps, replacing endogenous sequences iteratively until the endogenous chromosomes had been fully removed (Mitchell et al. 2017; Richardson et al. 2017; Zhang et al. 2017). The ability to design and “build” a chromosome from scratch for study would give researchers complete control over the extra genes present, the ability to insert fluorescent markers, create floxed alleles for lineage tracing and many other valuable methods used in genetic studies.

The presence of an extra chromosome may have a role in the pathogenesis of the trisomy aneuploidy syndromes in addition to phenotypes that arise from increased or decreased gene dosage. In the event that aneuploidy per se does confer phenotypes, this will affect how we undertake pre-clinical trials, perhaps shifting our current focus from ameliorating gene dosage effects, to approaches for removing the entire extra chromosome (in DS). This remains in the realm of science fiction currently, but science fiction has a habit of turning into science fact.

## Supplementary Information

Below is the link to the electronic supplementary material.

Supplementary file1 (XLS 527 KB)
Supplementary Table 1, the gene content of triplicated regions in aneuploid mouse models of Down syndrome.
